# Butyrate inhibits iILC2-mediated lung inflammation via lung-gut axis in chronic obstructive pulmonary disease (COPD)

**DOI:** 10.1186/s12890-023-02438-z

**Published:** 2023-05-12

**Authors:** Min Jiang, Zhiwei Li, Fengbo Zhang, Zheng Li, Dan Xu, Jing Jing, Fengsen Li, Jing Wang, Jianbing Ding

**Affiliations:** 1 State Key Laboratory of Pathogenesis, Prevention and Treatment of High Incidence Diseases in Central Asia, Xinjiang Medical University, Urumqi, 830011 Xinjiang China; 2 Xinjiang Key Laboratory of Respiratory Disease Research, Traditional Chinese Medical Hospital of Xinjiang Uygur Autonomous Region, No. 116, Huanghe Road, Urumqi, 830011 Xinjiang China; 3 Clinical Laboratory Center, People’s Hospital of Xinjiang Uygur Autonomous, Urumqi, 830001 Xinjiang China; 4 Department of Clinical Laboratory, the First Affiliated Hospital of Xinjiang Medical University, Urumqi, 830054 Xinjiang China; 5 Department of Immunology, College of Basic Medicine, Xinjiang Medical University, No. 4, Xinyi Road, Urumqi, 830011 Xinjiang China

**Keywords:** Chronic obstructive pulmonary disease (COPD), Acute exacerbation of COPD (AECOPD), Short chain fatty acids (SCFAs), Inflammatory ILC2s, Butyrate

## Abstract

**Background:**

The study investigated the effects and underlying mechanisms of intestinal flora metabolite butyrate on inflammatory ILC2 cells (iILC2s)-mediated lung inflammation in chronic obstructive pulmonary disease (COPD).

**Methods:**

Mouse models of COPD and acute exacerbation of COPD (AECOPD) were established. Flow cytometry was used to detect natural ILC2 cells (nILC2s) and iILC2s in lung and colon tissues. The 16s rRNA and GC-MS were used to detect microbial flora and short chain fatty acids (SCFAs) in feces. ELISA was used to detect IL-13 and IL-4. Western blot and qRT-PCR were used to detect the relative protein and mRNA levels, respectively. In vitro experiments were performed with sorted ILC2s from colon tissues of control mice. Mice with AECOPD were treated with butyrate.

**Results:**

The nILC2s and iILC2s in lung and colon tissues of AECOPD mice were significantly higher than control groups. The abundance of the flora *Clostridiaceae* was significantly reduced, and the content of SCFAs, including acetate and butyrate, was significantly reduced. The in vitro experiments showed that butyrate inhibited iILC2 cell phenotype and cytokine secretion. Butyrate treatment reduced the proportion of iILC2 cells in the colon and lung tissues of mice with AECOPD.

**Conclusions:**

The nILC2s and iILC2s in the colon tissues are involved in the course of COPD. Decreased *Clostridiaceae* and butyrate in AECOPD mice caused the accumulation of iILC2 cells in the intestines and lungs. Supplementation of butyrate can reduce iILC2 in the intestine and lung tissues. Our data may provide new ideas for prevention and treatment of COPD.

**Supplementary Information:**

The online version contains supplementary material available at 10.1186/s12890-023-02438-z.

## Background

Chronic obstructive pulmonary disease (COPD) is the third most common cause of death worldwide, which is characterized by lung inflammation, emphysema, and decreased lung function [1, 2]. Acute exacerbation of COPD (AECOPD), manifested by high airway and systemic inflammation, represents the main pathological process affecting the quality of life of COPD patients, which might lead to death [3]. Inflammation and immune response play key roles in the pathogenesis of COPD. The innate immune response is the basis of COPD inflammation, which mainly involves the macrophages (alveolar and interstitial), neutrophils, dendritic cells, natural killer cells, and innate lymphoid cells (ILCs) [4–8].

Studies have confirmed that type 2 innate lymphoid cell (ILC2) is involved in the COPD airway fibrosis, lung remodeling, and inflammation [9, 10]. Although the number of ILC2 cells in the lungs is limited, they are crucial in connecting innate immunity and adaptive immune responses and maintaining immune balance in the lungs [11]. Our previous studies have found that ILC2 was involved in the chronic inflammatory response caused by COPD Th1/Th2 immune disorder [12, 13]. ILC2 cells could regulate the Th2-type immune response through up-regulating the expression of MHCII, or secrete the cytokines of IL-13 and IL-4 through the notch-GATA3 pathway, providing a differentiated microenvironment for Th2 cells to promote Th2-type immune response, which in turn affects the acute exacerbation of inflammatory response in COPD [12, 13]. However, studies have shown that ILC2 cells can be divided into two distinct types, i.e., the inflammatory ILC2 cells (iILC2s, defined as Lin^−^CD45^+^CD127^+^ST2^−^KLRG1^hi^) and natural ILC2 cells (nILC2s, defined by Lin^–^CD45^+^CD127^+^ST2^+^KLRG1^int^) [14, 15]. Under pathological conditions, the iILC2s can be recruited from the distal end of the intestine to the lungs through the *lung-gut axis*, and the nILC2 cells in the lung (alone or in combination with these iILC2s) can participate in the responses of inflammatory signals [15, 16]. However, it is still unclear whether these iILC2s are involved in the development of COPD.

Short-chain fatty acids (SCFAs), the metabolites of intestinal flora, can be used as signaling molecules to affect the function of the intestinal immune system, thereby inhibiting the inflammatory responses in the distal lung [17]. As one of the SCFAs, butyrate can maintain the intestinal homeostasis by promoting the differentiation of the Treg cells [18, 19]. Moreover, butyrate can significantly inhibit the proliferation and activation of the murine ILC2 cells, both in vivo and in vitro, and reduce the production of cytokines [11]. In addition, butyrate can down-regulate the expression of GATA3 in ILC2 cells, inhibit the activity of ILC2 cells in the lungs, reduce the pulmonary airway hyperresponsiveness [17], and reduce the levels of metabolism, oxidative phosphorylation, and glycolysis pathways. Importantly butyrate is one of the microbial metabolites that interact most strongly with COPD-related host genes [20]. It has shown beneficial effects in the pathological conditions of lung diseases [21]. However, the mechanism by which butyrate regulates immune cells to participate in the COPD inflammatory response remains unclear.

In this study, the effects and underlying mechanisms of the intestinal flora metabolite butyrate in iILC2s-mediated lung inflammation of COPD were investigated. The changes of the nILC2s and iILC2s in COPD intestines and lung tissues were investigated, both in the in vivo and in vitro models. Moreover, based on the *lung-gut axis* theory, the effects of SCFAs (acetate and butyrate) on the colon ILC2 cells were also studied. The changes of the colon iILC2 in different stages of COPD and its relationship with pulmonary inflammation were investigated. Our findings may to provide new ideas for the prevention and treatment of COPD inflammation in clinic.

## Methods

### Animals

Totally, 75 SPF male C57BL/6J mice (6-week old, weighing 20 ± 2 g) were purchased from the Experimental Animal Center of Xinjiang Medical University. The breeding conditions were as follows: temperature, 22–25 ℃; relative humidity, 45-65%; and light and dark cycle, 12 h/12 h. All methods were performed in accordance with the relevant guidelines and regulations. The animal experiment procedures were approved by the Animal Protection and Welfare Committee of Xinjiang Medical University. The study is reported in accordance with ARRIVE guidelines.

### Animal grouping and construction of mouse model of COPD

After 1 week of acclimation, mice were randomly divided into the following groups: (1) the control group (n = 15), in which the animals were subjected to room air inhalation without cigarette smoke (CS) exposure; (2) the CS exposure group (n = 15), in which the animals were subjected to the CS exposure; and (3) the LPS group (n = 15), in which the CS model mice were treated with LPS (7.5 µg in 50 µL saline; Sigma-Aldrich, St. Louis, MO, USA). To establish the COPD mouse model, from day 1 to day 90, the animals were placed into the homemade glass fumigation box (60 × 40 × 30 cm^3^) for CS exposure (9 cigarettes/h, for 2 h each time, 2 times per day, and 6 days every week). The flow rate was set at about 0.5 L/min, and the smoke concentration was maintained at about 396 ± 5 mg/m^3^, as determined by the smoke detector (Nippon Kanomax 3521, Japan). LPS or saline was intranasally delivered to the CS-exposed mice on day 91 and day 105. The cigarettes used for the mouse model establishment were the commercial Xuelian brands (tar amount 12 mg, smoke nicotine amount 1.0 mg; the flue gas CO amount was 13 mg; Xinjiang Cigarette Factory, Urumqi, Xinjiang, China).

For butyrate treatment, the mice in the CS + LPS + butyrate group (n = 15) were given 150 mM sodium butyrate (pH 7.5) (Sigma-Aldrich, St. Louis, MO, USA) intragastrically for two weeks (from day 107 to day 120). For control, normal saline was given intragastrically to mice of CS + LPS (n = 15).

### Airway responsiveness measurement

After the establishment of COPD model, we measured the airway responsiveness to methacholine using the non-invasive pulmonary function instrument (Fine-Pointe NAM system; St. Paul, MN, USA). Briefly, conscious and spontaneously breathing mice were challenged with methacholine (Sigma-Aldrich, St. Louis, MO, USA) at indicated concentrations (i.e., 0.00, 3.125, 6.25, 12.50, 25.00, and 50.00 mg/mL). After each challenge, the enhanced exhalation interval (enhanced pause, Penh) was calculated.

### Sampling

All animals were subjected to lung function assessment before sacrifice on day 106. Metabolic cages were used to collect the fecal samples of each mouse (more than 50 mg per mouse). The mice were sacrificed with carbon dioxide. Briefly, the mice were placed in a euthanasia box (RC-1001-15 L; Shanghai Yuyan Instrument Co., Ltd., China) with a carbon dioxide filling rate of approximately 10–30% of the chamber volume per minute. When the mice stopped breathing, carbon dioxide filling was performed for another 1 min. The lung and colon tissues were harvested. The blood was collected from the abdominal aorta. Serum was isolated from the blood after centrifugation.

### HE staining

The left lung tissues were fixed in 4% neutral formaldehyde, routinely dehydrated, embedded in paraffin, and cut into 5-µm continuous sections. The sections were dewaxed with xylene and hydrated with gradient ethanol; stained with hematoxylin for 5–10 s, differentiated with 1% hydrochloric acid ethanol for 5–10 s, and stained with eosin for 1–2 min; were subjected to the gradient ethanol dehydration and xylene transparent treatment; and mounted with neutral resin. Histopathological observation was performed under a light microscope. Pulmonary histopathological scoring was performed based on the following 7 indicators: (1) epithelial shedding and erosion; (2) epithelial goblet cell hyperplasia; (3) Bronchial wall neutrophil infiltration; (4) Bronchial wall lymphocyte infiltration and lymphoid follicle formation; (5) Bronchial wall mononuclear and macrophage infiltration; (6) Bronchial wall eosinophil infiltration; and (7) Bronchial wall luminal exudation. The scoring standard was as follows: 0 point, no lesion; and 1, 2, and 3 points, mild, moderate, and severe abnormalities, respectively. The scores of each indicator were accumulated to obtain the pathological scores of each specimen.

### Bronchoalveolar lavage fluid (BALF) collection and cell counting

The mice were sacrificed with carbon dioxide, and the trachea was separated. Bronchoalveolar lavage was performed with 0.8 mL sterile PBS pre-cooled at 4 °C (repeated 3 times). The recovery rate of BALF was as high as 75%. After centrifugation at 1500 rpm at 4 °C for 10 min, the supernatant and precipitate were respectively collected. The precipitate was re-suspended in 100 µL sterile PBS, and the total numbers of cells, neutrophils, monocytes and lymphocytes were counted under a light microscope.

### 16 S rRNA gene sequencing

Total genome DNA was extracted from fecal samples using the CTAB (cationic detergent cetyl-trimethylammonium bromide) method. Fecal 16 S rRNA genes were analyzed via the Illumina MiSeq (CA, USA) to determine the intestinal microbiota composition. The 16 S rRNA/18SrRNA/ITS genes of distinct regions (16 S V4/16S V3/16S V3 -V4/16S V4-V5,18 S V4/18S V9, ITS1/ITS2, Arc V4) were amplified using the specific primers (e.g., 16 S V4: 515F806R, 18 S V4: 528 F-706R, 18 S V9: 1380 F-1510R, et al.) with the barcode. The DyNAmo SYBR green quantitative PCR kit (Finnzymes, Thermo Fisher Scientific, MA, USA) were used for the quantifying amplifications. Sequencing libraries were generated using the TruSeq® DNA PCR-Free Sample Preparation Kit (Illumina, USA). The library quality was assessed on the Qubit@ 2.0 Fluorometer (Thermo Scientific) and Agilent Bioanalyzer 2100 system. At last, the library was sequenced on the Illumina NovaSeq platform (Illumina, San Diego, CA, USA), and 250 bp paired-end reads were generated.

### Sequencing data analysis and bioinformatics analysis

The original off-machine data of high-throughput sequencing was initially screened according to the sequence quality. The sequences passing the quality preliminary screening were identified and assigned to corresponding samples according to the primer and barcode information. On the other hand, the questionable sequences such as chimeras were removed. The obtained sequence could be checked. Using the Uparse software (Uparse v7.0.1001; http://drive5.com/uparse/), the sequences obtained were merged and divided into operational taxonomic units (OTUs) based on the sequence similarity of 97%. The representative sequence of each OTU was used for classification status identification. According to the serial number standard of the sample with the least sequence, the abundance information of OTU was normalized. A heatmap was generated according to the relative abundance of the OTUs using R v3.1.1.

### Gas chromatography-mass spectrometry (GC-MS) of SCFAs

GC-MS was used to detect the content of SCFAs, including acetic acid, propionic acid, butyric acid, isobutyric acid, valeric acid, and isovaleric acid in mouse fecal samples. First, 50 mg fecal sample was accurately weighed, and 50 µL of 15% phosphoric acid, 10 µL of 75 µg/mL internal standard (isohexanoic acid), and 140 µL ether were added for homogenization for 1 min. After centrifugation at 4℃ at 12,000 rpm for 10 min, the supernatant was collected and tested with GC-MS. The GC-MS parameters were chromatographic column Agilent HP-INNOWAX capillary column (30 m*0.25 mm ID*0.25 μm); split injection; injection volume 1 µL; and split ratio 10:1. MS conditions were electron impact ionization source, SIM scanning mode, and electron energy 70 eV. The concentrations of each SCFA were calculated according to the standard curves.

### Flow cytometry

Flow cytometry was used to detect the ILC2 cells in the lung and colon tissues. Briefly, the mouse lung/colon single-cell suspension in PBS containing 1% BSA was obtained from lung/colon tissues after homogenization, digestion and centrifugation. The cell suspension (100 µL; cell concentration 1 × 10^6^ cells/mL) was incubated with the following antibodies (all from BD unless mentioned): the APC-CY7-CD45 (2D1), Lineage-FITC, CD3 (UCHT1), CD19 (HIB19), CD123 (7G3), CD11b (M1/70), CD11c (B-ly6), CD8 (RPA-T8), FceRI (AER-37 (CRA-1), CD14 (M5E2), CD4 (RPA-T4), CD56 (B159), PerCP-CY5.5-90.2 (30-H12, BioLegend, San Diego, CA, USA), PE-CY7-ST2 (DIH4), and PE-CD127 (SB/199), at room temperature for 30 min in dark. After washing with PBS twice, flow cytometry was performed to detect the ratio of Lin^−^CD45^+^CD127^+^ST2^+^KLRG1^int^ cells and Lin^−^CD45^+^CD127^+^ST2^−^KLRG1^hi^ cells with the cytometer (LSR II, BD, USA). The data were analyzed using the Kaluza software (Beckman Coulter, Inc).

### ILC2 cell sorting and treatment

Leukocyte cell suspension was prepared. Briefly, 30 mg of colon tissues were washed with pre-cooled PBS and then incubated in dissociation buffer (HBSS^−/−^ supplemented with 5mM EDTA and 10mM Hepes) at 37 °C for 15 min. After washing, the colon tissues were mince into fine pieces using scissors and incubated with digestion buffer (4% FBS, 1mM HEPES, 0.5 mg/mL collagenase, and 0.5 mg/mL DNase I) at 37 °C for 20 min. The supernatant was collected after filtration through a 70 μm cell strainer. After centrifugation at 500 g for 10 min, the cells were collected from the supernatant and then re-suspended in 5 mL of 40% Percoll (GE Healthcare). The 5mL 80% Percoll was added to the 40% Percoll containing the cells and centrifuged at 1300 g for 20 min at 20 °C. The opaque layer at the interface between the 40% and 80% Percoll was considered as leukocytes. Magnetic beads (STEMCELL, Vancouver, BC, Canada) were used to sort the colon ILC2 cells, with the sorting purity > 95%. The sorted colon ILC2 cells were inoculated onto the 96-well plate (500 cells/well), and cultured with the IMDM culture medium containing 10% FBS, 500 ng/mL IL-2 (200-02-100, peprotech), 500 ng/mL IL-25 (8134-IL-025, R&D), 500 ng/mL IL-33 (R&D), and 500 ng/mL TSLP (300-62-10, peprotech), for 5 days. Then, after washing twice to remove the cytokines, the colon ILC2 cells were treated with SCFAs. Briefly, 5 × 10^3^ ILC2 cells were inoculated onto the 96-well plate and were then treated with normal saline (control), acetate and butyrate (Sigma-Aldrich, St. Louis, MO, USA), at 2 µM, 20 µM, and 200 µM, respectively, for 6 h. The culture supernatant was collected for ELISA analysis.

#### Cell viability assay and apoptosis assay

Cell viability was detected by Cell Counting Kit-8. Briefly, 10 µL of CCK-8 was added to 100 µL cell suspension at 1 h before the end of culture. Absorbance was measured at 450 nm by spectrophotometer.

For apoptosis assay, 5 × 10^3^ colon ILC2 cells were inoculated onto the 96-well plate and were then treated with normal saline (control) or butyrate (Sigma-Aldrich, St. Louis, MO, USA), at 2 µM, 20 µM, and 200 µM, respectively, for 6 h. Then cells were harvested and stained with Annexin V-PE/7-AAD for 20 min. Cell apoptosis was analyzed by flow cytometry using the Beckman DXFLEX (Beckman Coulter, Inc).

### ELISA

The contents of IL-13 and IL-4 in the BALF and cell culture supernatant were measured with the ELISA kits (Lianke Biotechnology Co., Ltd., Hangzhou, Zhejiang, China). The absorbance (OD) values at 450 nm were measured with a microplate reader.

### Quantitative real-time PCR

Total RNA was extracted from colon ILC2s using Trizol LS (Takara, Tokyo, Japan). Then, the cDNA was synthesized from the total RNA using the Transcriptor first strand cDNA synthesis kit (Takara, Tokyo, Japan). The mRNA levels of *IL-13* and *IL-4* were detected with the quantitative real-time PCR on the BIO-RAD CFX96 detection system (Bio-Rad, Hercules, CA, USA)). Primers for these genes were as follows: IL-13, forward 5’-AATGGCAGCATGGTATGGAGCATC-3’ and revere 5’-GCAGAATCCGCTCAGCATCCTC-3’; IL-4, forward 5’-GCCTACAAAGCCCAGAGAGAACAC-3’ and reverse 5’-TTGTGCCTGTGGAACTGCTGTG-3’; and β-actin, forward 5’-CATGTACGTTGCTATCCAGC-3’ and 5’-CATGTACGTTGCTATCCAGC-3’. The PCR procedure was as follows: 95 °C for 5 min, followed by 40 cycles of 95 °C for 30 s, and 60 °C for 30s. The expression levels of the target genes were calculated with the 2^−ΔΔCT^ method. β-actin was used as internal reference.

### Western blot

Colon ILC2 cells were lysed with cold RIPA buffer (Thermo Fisher Scientific, Waltham, MA, USA) for protein extraction. Totally 25 µg protein was separated by 12% SDS-PAGE, which was then transferred onto the polyvinylidene fluoride membrane. The membrane was blocked with 5% nonfat milk at room temperature for 2 h, and further incubated overnight with rabbit anti-Arginase1 (anti-Arg1) (Sangon Biotech, Shanghai, China), rabbit anti-Killer cell lectin-like receptor G1 (KLRG1) (Abcam, Cambridge, MA, USA), rabbit anti-receptor B (IL-17RB) (Solarbio, Beijing, China), or mouse anti-β-actin antibodies (Abcam, Cambridge, MA, USA) at 4 °C. After washing, the membranes were incubated with the secondary antibody (Abcam, Cambridge, MA, USA) and finally processed using the enhanced chemiluminescence reaction kit (Cell Signaling Technology, Danvers, MA, USA). The relative expression levels were quantified using the Quantity One 1-D analysis software package (Bio-Rad, Hercules, CA, USA).

### Statistical analysis

Data were expressed as mean ± SEM. The Graphpad Prism 8 software package (Graphpad Software Inc., San Diego, CA, USA) was used for statistical analysis. One-way ANOVA was used to compare the data among multiple groups, followed by LSD-t test. P < 0.05 was considered as statistically significant.

## Results

### Lung inflammation is augmented in CS exposed mice challenged with LPS

**Fig. 1:**
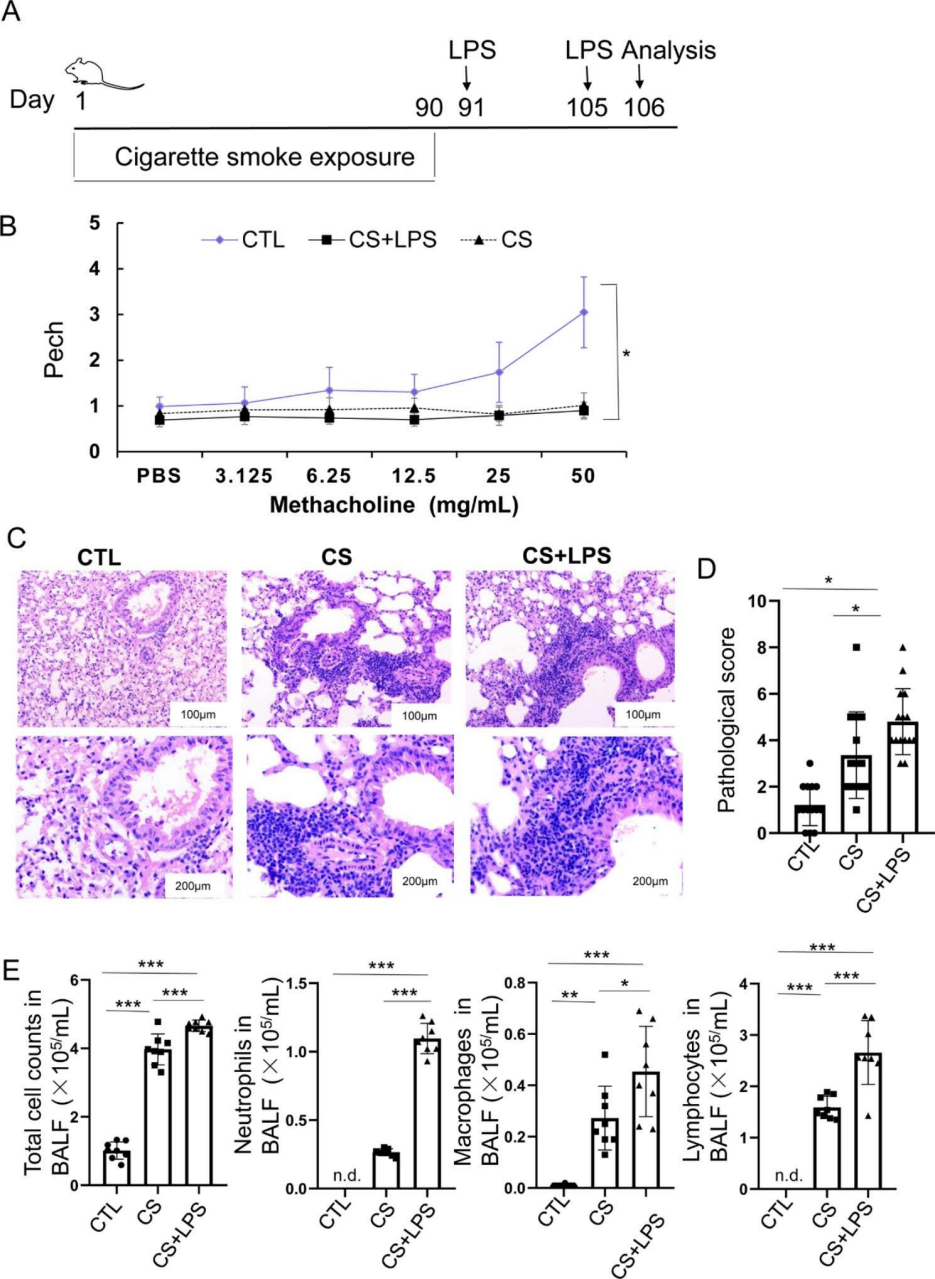
CS + LPS increases lung inflammation and decreases airway compliance in mice. (**A**) Study design for COPD model caused by cigarette smoke exposure and LPS-triggered COPD exacerbation model. Mice were exposed to cigarette smoke for 90 days with intranasal inhalation of LPS on days 91 and 105. (**B**) Penh value of mice challenged with increasing dose of methacholine (n = 15). (**C**) Lung sections were subjected to H&E staining. (n = 5). (**D**) Airway or peri-airway inflammation was assessed. (n = 5). (**E**) Numbers of total cells, neutrophils, macrophages and lymphocytes in mouse BALFs. *P < 0.05, **P < 0.01 and ***P < 0.001 (n = 5)

To study the acute exacerbation of lung inflammation in COPD, mice were exposed to CS for 90 days with intranasal inhalation of LPS on day 91 and day 105 (Fig. 1A). Penh, which represents pulmonary airflow resistance, was used to evaluate the airway hyperresponsiveness. Our results showed that after methacholine challenge, the mice in the control group had higher dynamic compliance, while the mice in the CS and CS + LPS groups had lower resistance (Fig. 1B), indicating that the pulmonary airflow of the CS and CS + LPS groups is obstructed (Fig. 1B). HE staining showed that, in the control group, the lung tissue structure was clear, with intact bronchial mucosal epithelium, and regularly arranged smooth muscle layer and submucosa (Fig. 1C). Moreover, the surrounding alveolar tissue structure was clear and complete. However, in the CS group, some of the bronchial wall was damaged; some of the bronchial mucosa ciliated columnar epithelium proliferated; and some showed atrophy and shedding. Moreover, the smooth muscle layer and submucosa structure were slightly disordered, and there were more chronic inflammatory cell infiltration around the bronchial wall. Some alveolar walls of the surrounding lung tissue were thickened, and partly consolidation was observed, with more chronic inflammatory cell infiltration in the interstitium, as well as enhanced expansion and rupture of some alveolar walls. Compared with the CS group, the LPS + CS treatment further aggravated the destruction of the bronchial wall, the degree of mucosal epithelial shedding, and the degree of chronic inflammatory cell infiltration. Acute inflammation cell infiltration was increased. The pathological score in CS + LPS group was significantly higher than the control and CS exposure groups (Fig. 1D).

The total numbers of cells, neutrophils, monocytes, and lymphocytes in the BALF were further analyzed. Our results showed that, when treated with LPS, the numbers of total cells, neutrophils cells, monocytes, lymphocytes in BALF from the CS and CS + LPS-stimulated mice were significantly increased (Fig. 1E).

These results suggest that CS can promote the inflammatory responses in the lungs of mice, and the LPS treatment further aggravates the inflammatory responses.

### The proportions of nILC2 and iILC2 in the lung tissues are increased in CS exposed mice challenged with LPS

**Fig. 2:**
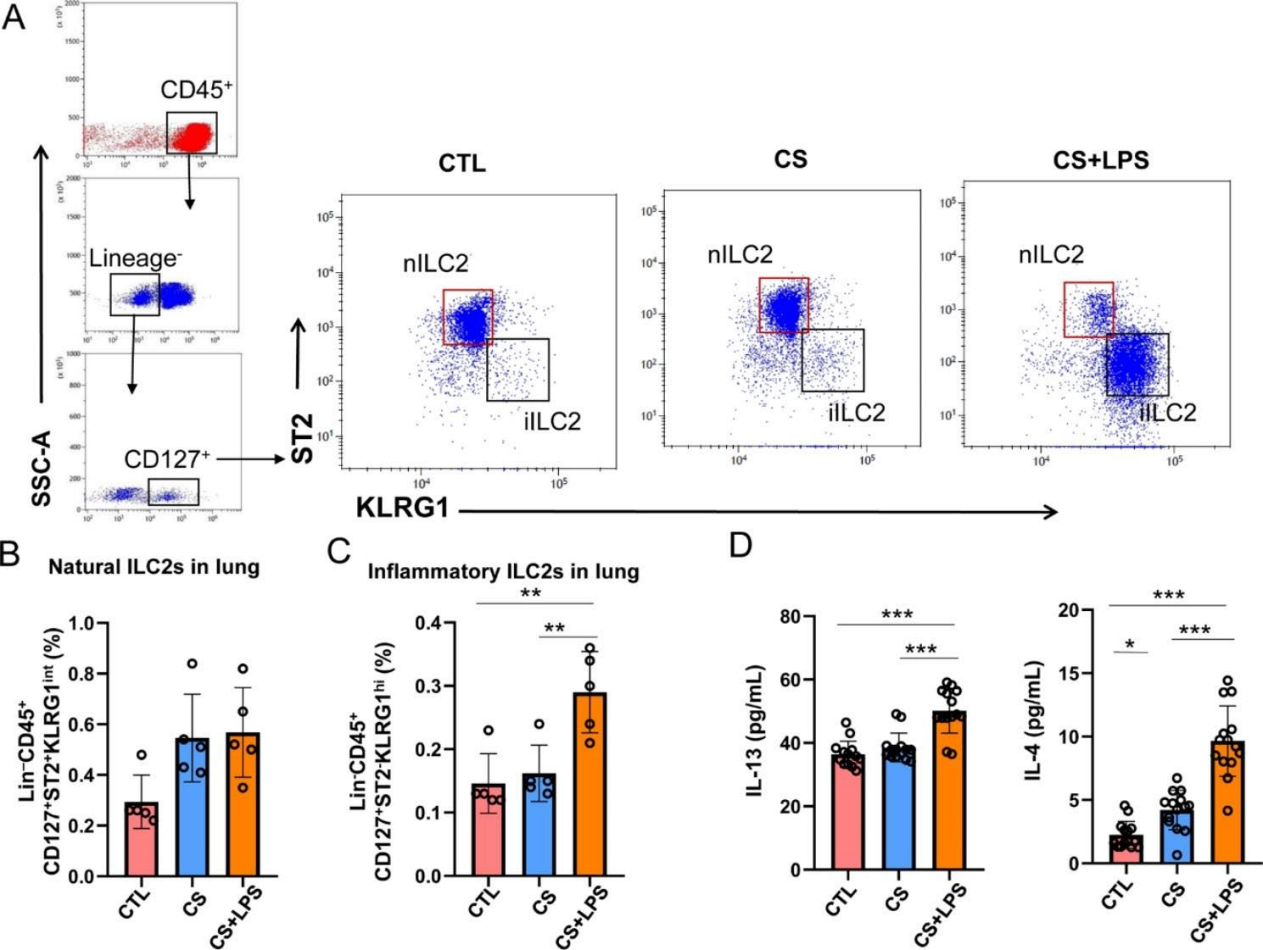
CS + LPS increases nILC2s and iILC2s in lungs, and IL-13/IL-4 in BALF. (**A**) Flow cytometry analysis of iILC2s (Lin^−^CD45^+^CD127^+^ST2^−^KLRG1^hi^) and nILC2s (Lin^–^CD45^+^CD127^+^ST2^+^KLRG1^int^) in the whole lung. The representative gates in each group were exhibited. (**B**) Percentage of nILC2s in these groups. (**C**) Percentage of iILC2s in these groups. (**D**) ELISA was used to detect the concentrations of cytokines IL-13 and IL-4 in BALF. *P < 0.05, **P < 0.01 and ***P < 0.001 (n = 5)

To study the changes of nILC2s and iILC2s in different courses of COPD, flow cytometry was used to detect these cells in the lung tissues in the CS and LPS-triggered exacerbation model mice. The gating strategy was shown in Fig. 2A. A significant increase was observed in iILC2s (Fig. 2C) in the lung tissue of the CS + LPS group, but not in nILC2s (Fig. 2B). Additionally, ELISA showed high expression levels of IL-13 and IL-4 in the BALF of mice in the LPS-triggered exacerbation model group (Fig. 2D). These findings indicate that, during the development of AECOPD, the proportion of iILC2s would be significantly increased in the lungs, and the expression levels of its related cytokines IL-13 and IL-4 would be significantly increased.

### The proportion of iILC2s is increased in the colon tissue

**Fig. 3:**
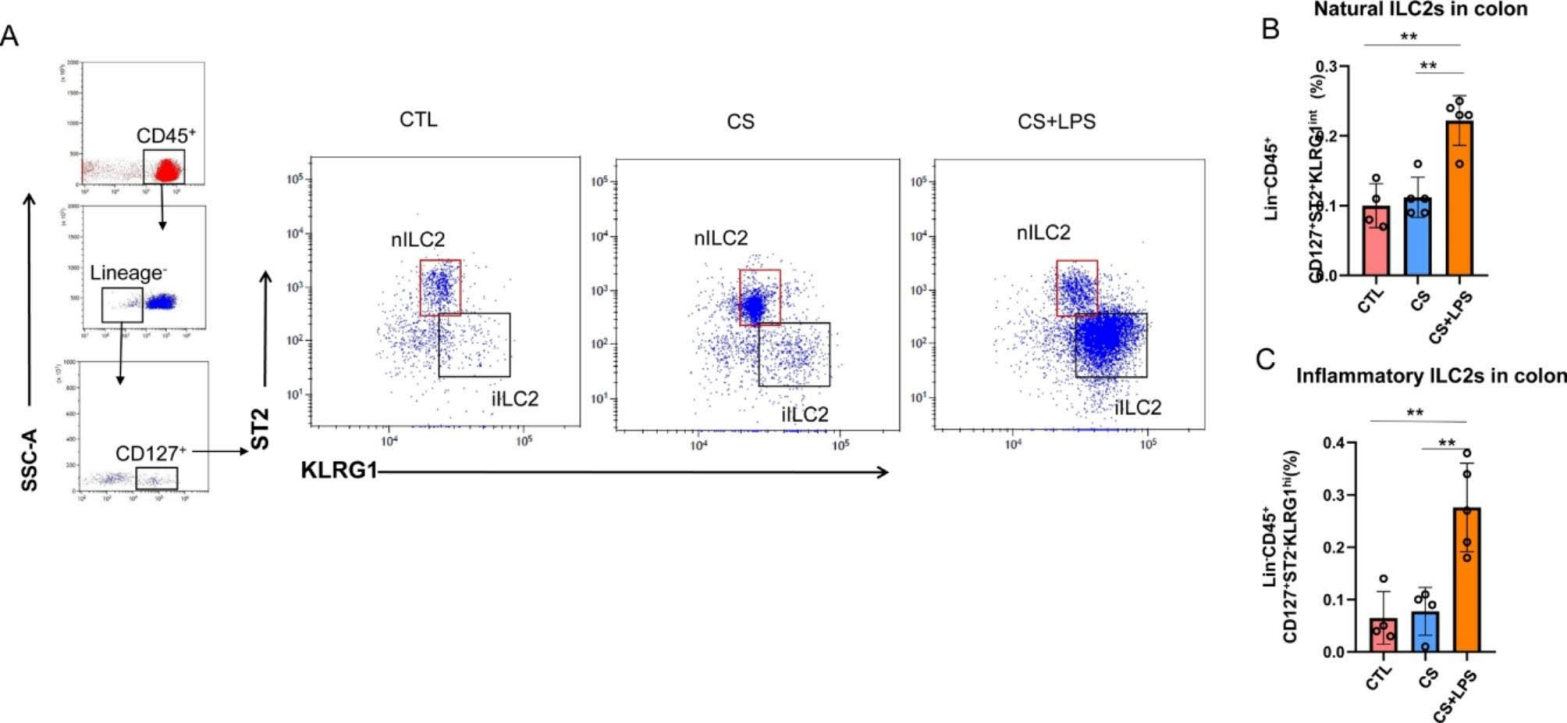
The nILC2s and iILC2s in the colon tissue of mice with AECOPD induced by LPS. (**A**) Flow cytometry analysis of iILC2s (Lin^−^CD45^+^CD127^+^ST2^−^KLRG1^hi^) and nILC2s (Lin^–^CD45^+^CD127^+^ST2^+^KLRG1^int^) in the colon tissue. The representative gates in each group were exhibited. (**B**) Percentage of nILC2s in these groups. (**C**) Percentage of iILC2s in these groups. *P < 0.05, and **P < 0.01 (n = 5)

Similarly, flow cytometry showed that, compared with the control, the nILC2s in the colon tissue was significantly increased in the CS + LPS group (Fig. 3A and B). Moreover, the iILC2s in the colon tissue of the CS + LPS group were significantly higher than the control and CS groups (Fig. 3C). These results indicate that the nILC2s and iILC2s in the colon tissues might be involved in the course of COPD. The high proportion of nILC2s and iILC2s in the colon tissues of mice in the LPS-triggered exacerbation model group further suggests that the nILC2s in the colon tissues may be transformed into iILC2s.

### Intestinal flora and SCFAs results

**Fig. 4:**
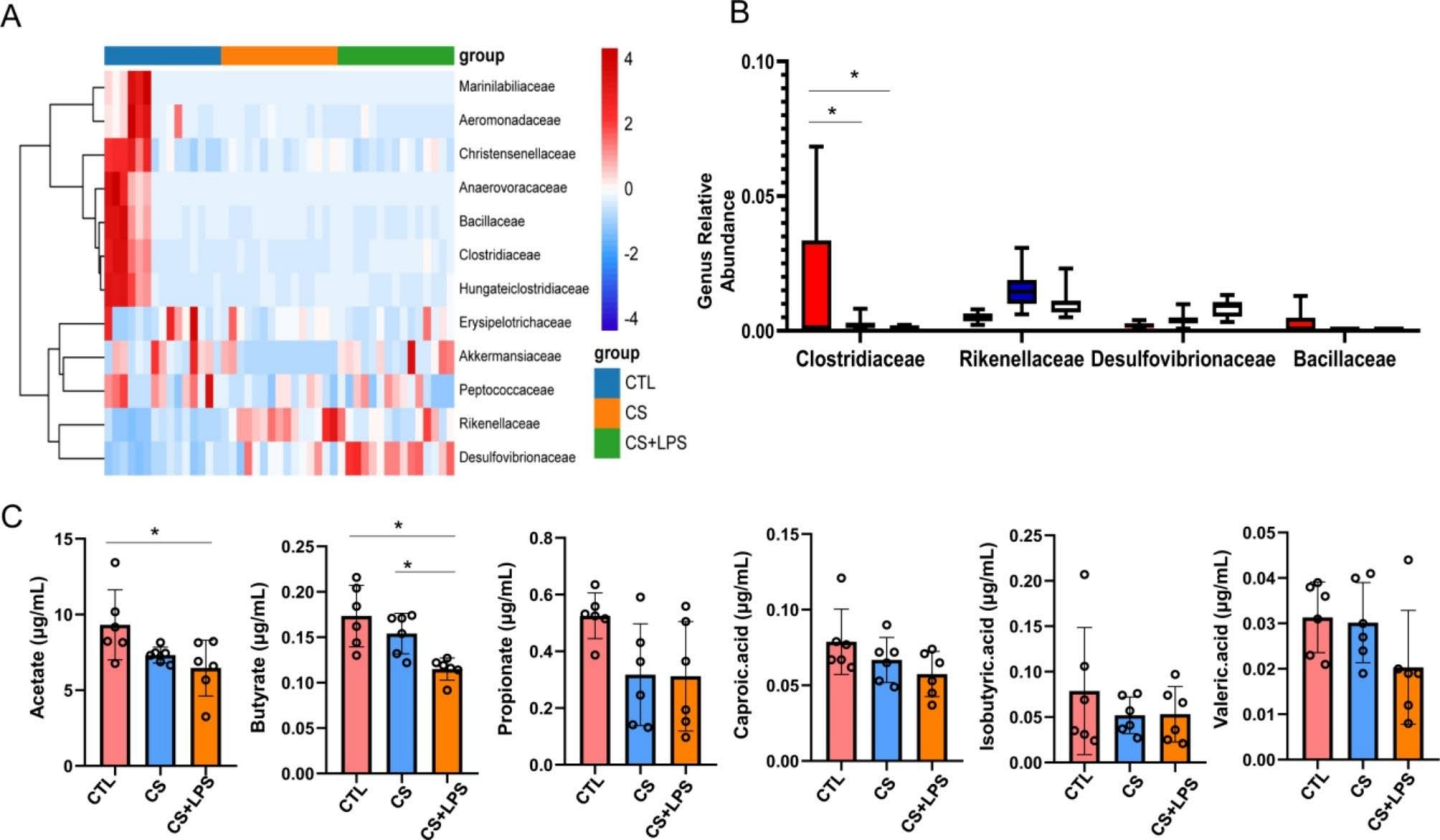
Abundance of intestinal flora in mice with AECOPD and COPD, and SCFA concentrations. (**A**) Heat map showing genus abundance by OUT count in fecal samples by 16 S V4 profiling. (**B**) Average proportions of most abundant bacterial genera. (**C**) Amounts of acetate, propionate, and butyrate in fecal samples analyzed by GC-MS. *P < 0.05 (n = 15)

The 16 S rRNA gene sequencing was carried out, and the composition of the intestinal bacterial community was analyzed and compared between the control, CS, and CS + LPS groups. Figure 4 A showed the heat map of each group. The proportions of *Clostridiaceae*, which could produce SCFAs, in the intestines of mice in the CS and CS + LPS groups were significantly reduced (Fig. 4B).

The SCFAs content in the feces of mice were further measured with GC-MS. Our results showed that the concentration of acetate in CS group and that of butyrate in the CS and CS + LPS group was deceased than control group (*P* < 0.05) (Fig. 4C). However, the concentration of other SCFAs was not significantly changed.

These results suggest that the decreased *Clostridiaceae* abundance in the intestines of mice in the CS and CS + LPS groups would decrease the concentration of butyrate, especially in the CS + LPS group. We believe that the decrease of butyrate in the intestine of mice in the CS + LPS group may be related to the acute exacerbation of COPD.

### Butyrate inhibits cytokine production by ILC2s in vitro

**Fig. 5:**
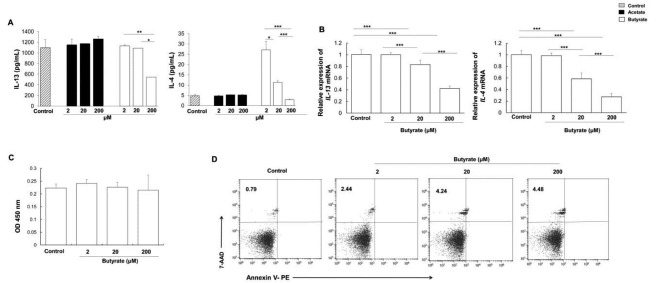
Butyrate suppresses iILC2 function in vivo. ILC2s were purified from the colon tissue of control mice. After proliferating in vitro, 5 × 10^3^ cells/well, treated in vitro with 2-200 µM SCFAs for 48 h. (**A**) IL-13 and IL-4 secretion in supernatant by activated ILC2 measured by ELISA after SCFA treatment. (**B**) The mRNA levels of *IL-13* and *IL-4* were tested by quantitative real-time PCR. (**C**) Cell viability assessed by CCK-8 assay. (**D**) Representative flow diagram showing PE and Annexin V positive ILC2s after 6-hour treatment. *P < 0.05, **P < 0.01 and ***P < 0.001 (n = 5)

To determine whether SCFAs can regulate the ILC2 function, the effects of acetate and butyrate on type 2 cytokine production by ILC2s were first investigated. The sorted gut ILC2s were treated with acetate and butyrate. Our results showed that butyrate, but not acetate, markedly reduced the IL-13 and IL-4 levels in the supernatant of cultured ILC2s, in a dose-dependent manner (Fig. 5A). Furthermore, quantitative real-time PCR showed significant reduction in the mRNA expression levels of both *IL-13* and *IL-4*, after 6-h treatment with butyrate (Fig. 5B). Our results showed that butyrate suppressed the secretion of IL-13 and IL-4 in the ILC2s cultured in vitro. These findings suggest that butyrate may inhibit the inflammatory function of ILC2s.

To investigate the effects of butyrate on the cell viability of ILC2 cells in vitro, CCK-8 assay was performed. The results revealed that butyrate did not significantly affect the cell viability of ILC2 cells at a concentration of 200 µM or lower (Fig. 5C). We further stained ILC2s with Annexin V-PE and found that the percentages of cells undergoing late apoptosis/necrosis (Annexin V^+^7-AAD ^+^) only increased slightly (< 5% at 200 µM) (Fig. 5D), and is therefore unlikely to cause the marked decrease in mRNA and protein levels of IL-13 and IL-4.

### Butyrate inhibits iILC2s transformation of ILC2s cells

**Fig. 6:**
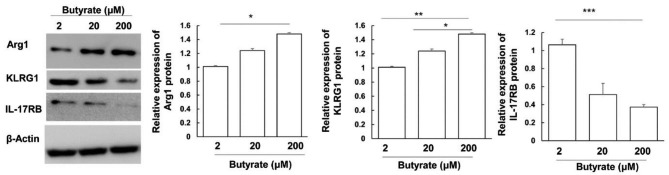
Butyrate inhibits the inflammatory transformation of ILC2 cells in vitro. Protein expression levels of Arg1, KLRG1 and IL-17RB were determined by Western blot analysis. Representative and quantitate Western blot results of Arg1, KLRG1 and IL-17RB in each group were shown. *P < 0.05, **P < 0.01 and ***P < 0.001 (n = 5)

The iILC2s highly express KLRG1 and IL-17RB, but almost do not express Arg1, while the nILC2s express high Arg1 and low IL-17RB [22]. To further study the effects of butyrate on the ILC2s, the expression levels of Arg1, KLRG1, and IL-17RB in the ILC2s treated with butyrate in vitro were assessed with Western blot. Our results showed that, compared with the 2 mM butyrate treatment group, the relative expression level of Arg1 in the 200 mM butyrate treatment group was significantly increased (*P*<0.05) (Fig. 6). Compared with the 2 mM and 20 mM butyrate treatment groups, the relative expression level of KLRG1 in the 200 mM butyrate treatment group was significantly decreased (*P*<0.05). Compared with the 2 mM butyrate treatment group, the relative expression level of IL-17RB in the 200 mM butyrate treatment group was significantly reduced (*P*<0.05) (Fig. 6). These results suggest that butyrate may reduce the transformation of iILC2s cells, in a dose-dependent manner.

### The proportions of iILC2s in the colon and lung tissues of mice with AECOPD are reduced after butyrate treatment

**Fig. 7:**
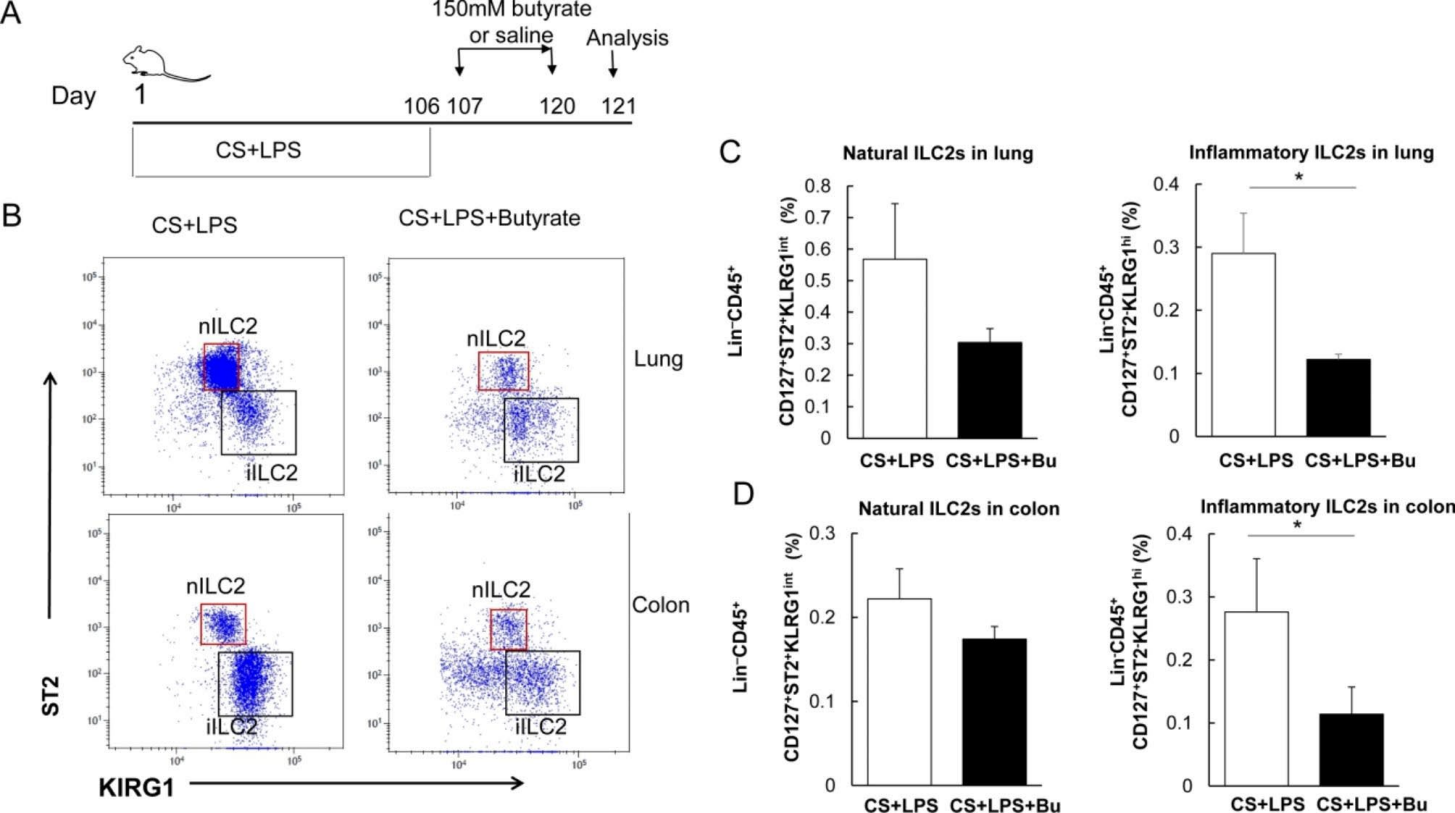
Changes of iILC2 proportion in the colon and lung tissues in mice with AECOPD after butyrate treatment in vivo. (**A**) Study flowchart for butyrate treatment in mice. (**B**) Flow cytometry analysis of iILC2s (Lin^−^CD45^+^CD127^+^ST2^−^KLRG1^hi^) and nILC2s (Lin^–^CD45^+^CD127^+^ST2^+^KLRG1^int^) in the lung and colon tissues. The representative gates in each group were exhibited. (**C**) Percentage of nILC2s and iILC2s in lungs. (**D**) Percentage of nILC2s and iILC2s in colons. *P < 0.05 (n = 5)

The effects of butyrate on acute exacerbation of lung inflammation in mice were investigated. The mice in the CS + LPS group were given 150 mM butyrate continuously for two weeks (Fig. 7A). Then, the nILC2s and iILC2s in the lungs and colon tissue were analyzed and compared. Our results showed that the proportions of iILC2s in the colon and lung tissues of mice treated with butyrate were significantly reduced (Fig. 7B and D). Therefore, we suppose that butyrate may inhibit the inflammatory transformation of colon tissue ILC2s, and reduce the colon iILC2 reserve, which may ultimately decline the accumulation of iILC2s in the lungs of COPD and reduce the inflammatory response in the lungs.

## Discussion

In this study, the changes and functions of nILC2s and iILC2s in the lungs and colons of AECOPD induced by CS exposure and LPS were investigated. Our results showed that the airway and lung inflammation was increased in the LPS-induced AECOPD, with increased iILC2s. These changes may be related to the decreased amount of *Clostridiaceae* in the intestine and the decreased butyrate concentration, which ultimately increase the colon iILC2s.

COPD is a chronic respiratory disease characterized by progressive, incompletely reversible airflow limitation and persistent airway response [23]. AECOPD refers to the deterioration of the original respiratory symptoms of patients with COPD [24]. AECOPD is usually induced by the viral infections and bacterial infections in the respiratory tract. Non-typeable *Haemophilus influenzae* is a common gram-negative bacterium that causes acute exacerbations in patients with AECOPD [25]. Gram-negative bacteria contain LPS, also known as endotoxin, which would be recognized by the host’s innate immune receptors and subsequently cause inflammation [26]. Therefore, in our study, LPS was used to induce COPD exacerbation caused by long-term CS exposure. We found that mice exposed to smoke for 3 months showed COPD-like characteristics, such as increased inflammation in the airways and lungs. The above-mentioned characteristics in CS-exposed mice challenged with LPS were sharply increased, indicating that the mouse model of COPD with acute exacerbation was successfully established.

Recent research has suggested that the ILC2s can be divided into two distinct types, i.e., the nILC2s and iILC2s [27]. In this study, we observed the changes of nILC2 and iILC2 in the lungs during COPD. Our results showed that the nILC2s and iILC2s in the lung tissue of mice with AECOPD were increased, and the concentrations of IL-13 and IL-4 cytokines were also significantly increased. In the experiments against *N. brasiliensis*, it has been shown that, after 5 days of infection, the IL-25 activated iILC2s to highly express IL-13, IL-5 and IL-4, and only the iILC2s expressed the Th2 cytokines [28]. Moreover, after infection for 8–10 days, the nILC2s in the lung were significantly increased, with high expression of IL-13 and IL-5, as well as a small amount of IL-4 [28]. IL-13 and IL-4 can promote the lung recruitment of eosinophils, neutrophils, and lymphocytes, and regulate the Th1/Th2 immune balance, leading to persistence of pulmonary inflammation [29, 30]. Hodge et al. [31] have found ILC2s in the lung tissue of COPD patients, and Wu et al. [32] have also shown increased ILC2s in the peripheral blood of COPD patients. Our previous study [12, 13] has shown that the numbers of ILC2 cells were increased in the peripheral blood of patients with AECOPD, and the Th2-type immune response was also increased. Silver et al. [33] have found that the numbers of ILC2s in the lungs of animal models of AECOPD caused by viral infection were decreased, and the transformation of ILC2s into ILC1s would result in AECOPD [33]. The iILC2s can be transformed into the nILC2s to complement the lung nILC2 cells [14]. It has also been confirmed that nILC2s in the lungs are tissue-resident cells [34]. However, after a parasite infection, the significantly increased nILC2s in the lungs are not only due to the self-renewal during inflammation, but the iILC2s may be recruited from other parts of the lungs, which could be neutralized and transformed into the nILC2s [34]. Therefore, we speculate that the increased nILC2s in the lungs of mice with AECOPD may be transformed by the iILC2s.

The phenotype and functional plasticity of ILC2s are affected by the homeostasis of the intestinal flora [35]. Our results in this study showed that, in the CS exposure group, the *Clostridiaceae*, and *Hungateiclostridiaceae* flora in the intestines were significantly reduced. It has been reported that the intestinal flora of patients with COPD changes drastically [36], among which *Clostridiaceae* is related to the production of SCFAs [17]. SCFAs play important roles in immune regulation and pulmonary inflammation. By regulating the activity of ILC2s and ILC3s with SCFAs, the optimal number of ILCs can be maintained in the peripheral tissues during infection and inflammation [37]. The changes in the SCFAs content in the feces were then investigated in this study. Our results showed that the acetate and butyrate in the intestines of AECOPD mice were significantly reduced, suggesting that the reduction of SCFAs may lead to aggravation of COPD. It is shown that the patients with COPD had intestinal dysfunction, with decreased concentration of acetate in plasma, which worsened as the disease severity increased [38]. Inhalation of butyrate can effectively alleviate lung inflammation [21]. In addition, increasing dietary fiber intake can reduce airway inflammation of COPD [39]. Experimental study in mice has shown that the intestinal diet based on whey peptides could increase the SCFAs in mice, and reduce the elastase-induced emphysema, slowing down the lung progression [40]. Therefore, the decreased SCFA contents in the intestines of COPD and AECOPD mice would increase the lung inflammation.

Studies have confirmed that SCFAs (such as acetate, propionate and butyrate) can promote the differentiation of the Treg cells [18, 41]. In addition, SCFAs can promote the differentiation of naive T cells into the Th1- and Th17-type effector cells [42], but SCFAs can inhibit the Th2 immune response [43]. SCFAs can also inhibit the function of ILC2s and reduce the expression levels of inflammatory factors [17, 37, 44]. Our results showed that butyrate significantly reduced the secretion of IL-13 and IL-4 by ILC2s cultured in vitro. Studies [15, 16] have shown that nILC2s highly express Arg1 and CD90, but rarely express IL-17RB, and highly secrete IL-13 and IL-5. However, the iILC2s highly express IL-17RB and KLRG1, rarely express Arg1, and slightly express IL-4. Moreover, nILC2s and iILC2s can be transformed into each other [15, 45]. Further results of our study revealed that butyrate inhibited the expression of IL-17RB and KLRG1 in ILC2s cultured in vitro while increased the expression of Arg1. Therefore, our results suggest that butyrate may inhibit iILC2 cell transformation, thereby inhibiting its cellular function in the course of COPD.

Due to the ability of colonic epithelial cells to take up large amounts of SCFAs under normal circumstance, the total amounts of SCFAs in the intestinal lumen are about 100–150 mM, while the SCFAs in the bloodstream are very low (usually 0.1-1 mM) [46]. Therefore, in this study, AECOPD mice were treated with 150 mM butyrate for 2 weeks, as previously described [15]. Our results of flow cytometry showed that butyrate treatment for two weeks significantly reduced iILC2s in the colon of AECOPD mice. Meanwhile, both iILC2s and nILC2s in the lungs of AECOPD mice were significantly reduced. At present, the progenitors of iILC2s have not yet been identified. However, many studies [14, 47] have shown that the intestine is the main source of iILC2s. Under pathological conditions, the iILC2s in the intestine can pass through the endothelium of the villi lymphatic vessels, and enter the blood circulation, thereby achieving the recruitment from the intestine to the lungs through the *lung-gut axis* [15, 16]. Based on the theory of the *lung-gut axis*, our results suggest that the increased iILC2s in the lungs of mice in the acute exacerbation period may be related to the increased iILC2s in the intestine. The treatment with butyrate may inhibit the inflammatory conversion of ILC2s in the colon into iILC2s, thereby reducing iILC2 reserve in the intestine and their distant recruitment to the lungs. The reduced iILC2s in the lungs may affect result in less conversion into nILC2s, finally allowing ILC2s to regulate the lung adaptive immune responses of COPD. However, we only obtained indirect evidence on the recruitment of iILC2 from the gut to the lungs. To obtain the direct evidence of distal lung metastasis of intestinal iILC2, the heterologous symbiosis experiments are needed.

It has been shown that metformin reduces the respiratory deterioration rate and improves the quality of life of patients with asthma-COPD overlap [48]. Interestingly, metformin also induces butyrate producing taxa of the gut microbiome [49], and the SCFAs and the butyrate producing taxa in the gut can improve lung health. Therefore, the mechanism of SCFAs in patients with asthma-COPD overlap and patients with COPD should be separately considered in the future.

## Conclusions

**Fig. 8:**
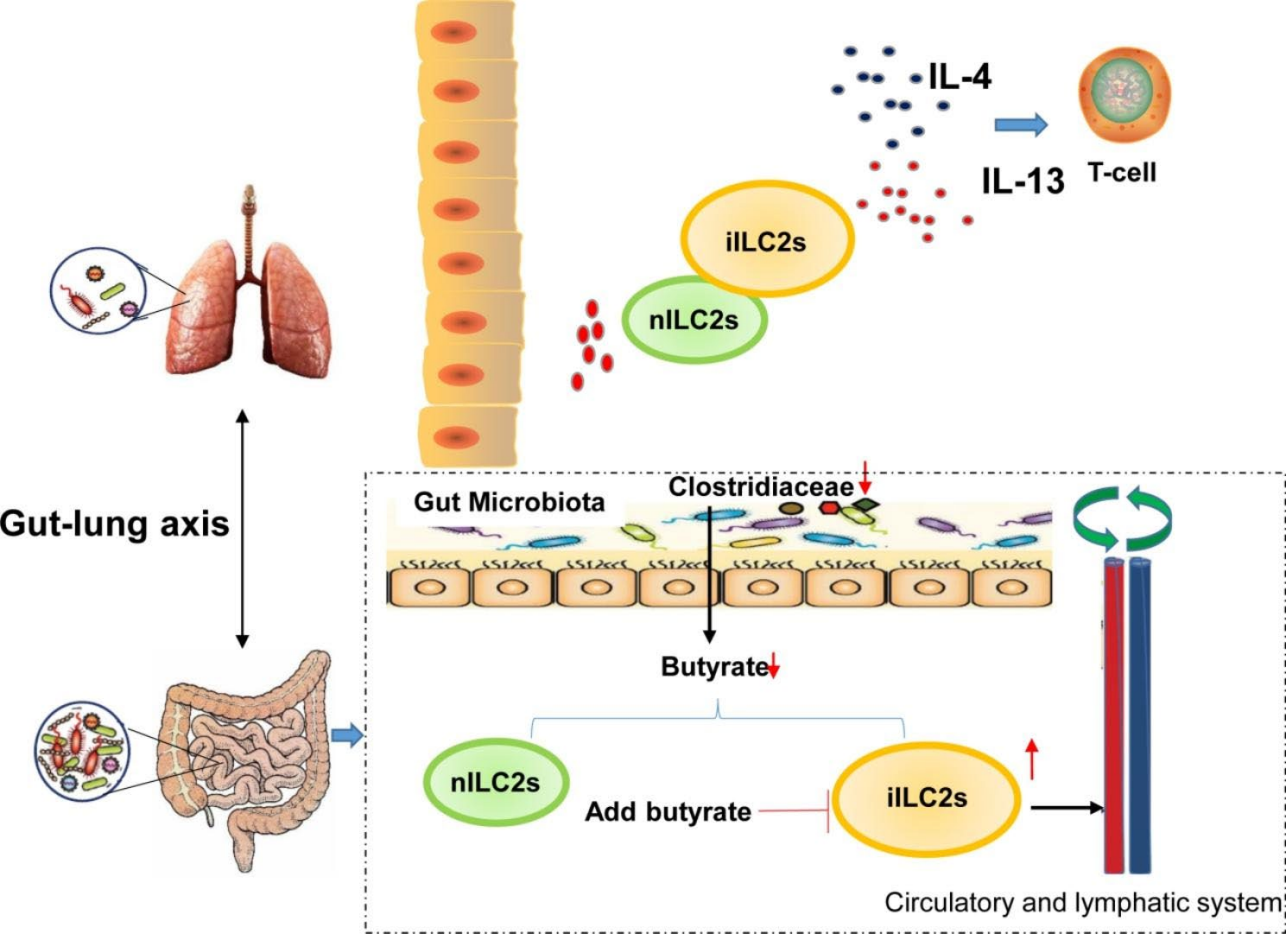
Schematic diagram showing the involvement of nILC2s, iILC2s, intestinal flora and butyrate in AECOPD. Acute exacerbation of the intestinal flora *Clostridiaceae* in COPD mice decreased butyrate, which ultimately promoted the recruitment of intestinal iILC2 into the lungs and enhanced type 2 cytokines (IL-4 and IL-13), participating in the COPD inflammatory responses

In conclusion, our results showed that there were nILC2s and iILC2s in the lungs of COPD mice. The proportion of iILC2s tended to be increased as the degree of inflammation increases. Our results also showed that, as the disease worsened, the intestinal flora of COPD mice became imbalanced, and the abundance of *Clostridiaceae* was decreased. Subsequently, the decreased butyrate was detected in SCFAs. The in vitro cell culture experiments found that butyrate inhibited the expression of iILC2 characteristic markers and inhibited the expression of IL-13 and IL-4 cytokines. However, when COPD exacerbated acutely, possibly due to the imbalance of the *Clostridiaceae*, butyrate was decreased, thus losing the inhibitory effects on the inflammatory transformation of ILC2s and increasing iILC2s in the intestine. Based on the theory of the *lung-gut axis*, we speculate that the iILC2s in the intestine may be recruited to the lungs through the *lung-gut axis*, and eventually affect the inflammation of the lungs (Fig. 8).

## Electronic supplementary material

Below is the link to the electronic supplementary material.

Supplementary Material 1

## Data Availability

The 16 S rRNA gene sequencing data were deposited in NCBI with accession number of PRJNA779799 (https://www.ncbi.nlm.nih.gov/sra/PRJNA779799).

## Abbreviations

COPD: chronic obstructive pulmonary disease
AECOPD: acute exacerbation of COPD
ILCs: innate lymphoid cells
ILC2: type 2 innate lymphoid cell
nILC2s: natural ILC2 cells
iILC2s: inflammatory ILC2 cells
SCFAs: short chain fatty acids
