# Supplementary figures and images for: Butyrate inhibits iILC2-mediated lung inflammation via lung-gut axis in chronic obstructive pulmonary disease (COPD)
Source: BMC Pulm Med. 2023 May 12;23:163. doi: 10.1186/s12890-023-02438-z (PMC10182695; doi:10.1186/s12890-023-02438-z)

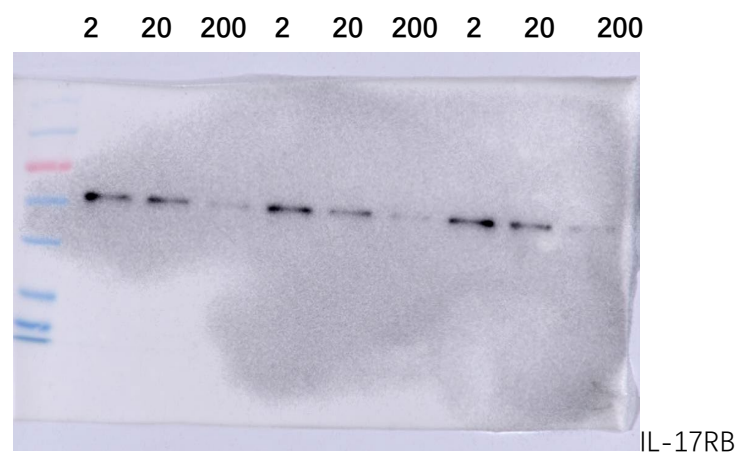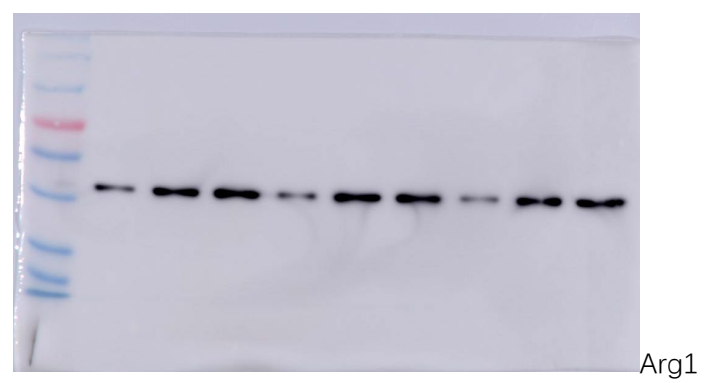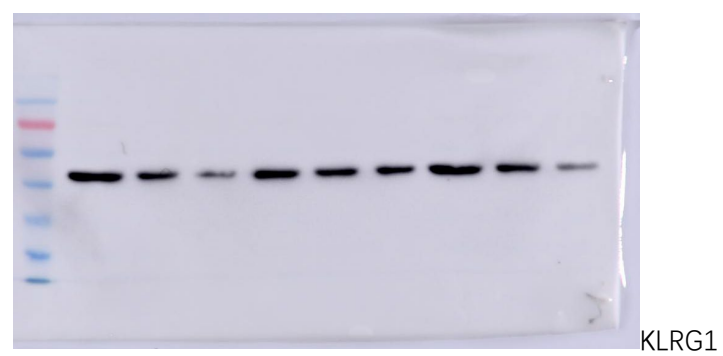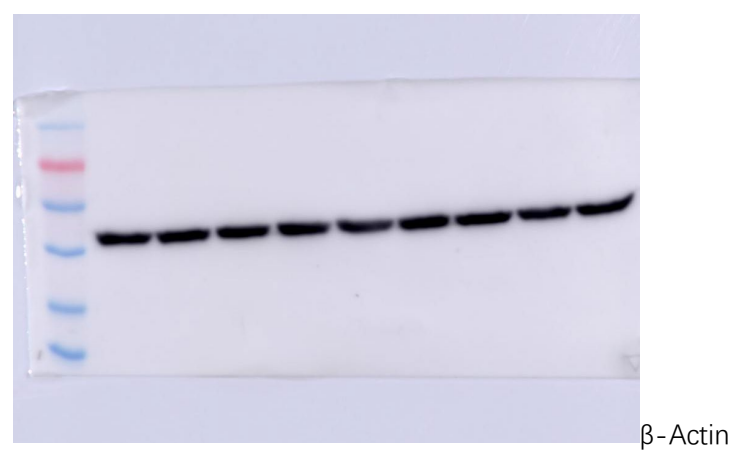

Supplement: Supplementary file 1 — Supplementary Material 1 [file 12890_2023_2438_MOESM1_ESM.pdf]
